# *Streptococcus equi* subsp. *zooepidemicus* Invades and Survives in Epithelial Cells

**DOI:** 10.3389/fcimb.2017.00465

**Published:** 2017-11-06

**Authors:** Bolette Skive, Manfred Rohde, Gabriella Molinari, Thomas Hartig Braunstein, Anders M. Bojesen

**Affiliations:** ^1^Veterinary Clinical Microbiology, Department of Veterinary and Animal Sciences, Faculty of Health and Medical Sciences, University of Copenhagen, Frederiksberg, Denmark; ^2^Central Facility for Microscopy, Helmholtz Centre for Infection Research, Braunschweig, Germany; ^3^Core Facility for Integrated Microscopy, Department of Biomedical Sciences, Faculty of Health and Medical Sciences, University of Copenhagen, Copenhagen, Denmark

**Keywords:** *Streptococcus equi* subsp. *zooepidemicus*, cell infection assay, immunofluorescence microscopy, scanning electron microscopy, intracellular survival, quantitative analysis of immunofluorescence data, equine endometritis

## Abstract

*Streptococcus equi* subsp. *zooepidemicus* (*S. zooepidemicus*) is an opportunistic pathogen of several species including humans. *S. zooepidemicus* is found on mucus membranes of healthy horses, but can cause acute and chronic endometritis. Recently *S. zooepidemicus* was found able to reside in the endometrium for prolonged periods of time. Thus, we hypothesized that an intracellular phase may be part of the *S. zooepidemicus* pathogenesis and investigated if *S. zooepidemicus* was able to invade and survive inside epithelial cells. HEp-2 and HeLa cell lines were co-cultured with two *S. zooepidemicus* strains (1-4a and S31A1) both originating from the uterus of mares suffering from endometritis. Cells were fixed at different time points during the 23 h infection assay and field emission scanning electron microscopy (FESEM) was used to characterize adhesion and invasion mechanisms. The FESEM images showed three morphologically different types of invasion for both bacterial strains. The main port of entry was through large invaginations in the epithelial cell membrane. Pili-like bacterial appendages were observed when the *S. zooepidemicus* cells were in close proximity to the epithelial cells indicating that attachment and invasion were active processes. Adherent and intracellular *S. zooepidemicus*, and bacteria in association with lysosomes was determined by immunofluorescence staining techniques and fluorescence microscopy. Quantification of intracellular bacteria was determined in penicillin protection assays. Both *S. zooepidemicus* strains investigated were able to invade epithelial cells although at different magnitudes. The immunofluorescence data showed significantly higher adhesion and invasion rates for strain 1-4a when compared to strain S31A1. *S. zooepidemicus* was able to survive intracellularly, but the survival rate decreased over time in the cell culture system. Phagosome-like compartments containing *S. zooepidemicus* at some stages fused with lysosomes to form a phagolysosome. The results indicate that an intracellular phase may be one way *S. zooepidemicus* survives in the host, and could in part explain how *S. zooepidemicus* can cause recurrent/persistent infections. Future studies should reveal the ability of *S. zooepidemicus* to internalize and survive in primary equine endometrial cells and during *in vivo* conditions.

## Introduction

*Streptococcus equi* subsp. *zooepidemicus* (*S. zooepidemicus*) is a Gram-positive, β-hemolytic coccus belonging to the Lancefield group C. It is an opportunistic pathogen for both humans and a broad range of animal species including horses, dogs, and pigs. It can cause severe diseases as pneumonia, septicemia, or meningitis (Pesavento et al., 2008; Byun et al., 2009; Blum et al., 2010; Eyre et al., 2010; Pelkonen et al., 2013). However, *S. zooepidemicus* can have a slow onset and cause localized infections as arthritis, local abscessation, and pericarditis, presumably spread hematogenously (Friederichs et al., 2009; Pelkonen et al., 2013), or have a chronic phase, as seen in an outbreak in chickens (Bisgaard et al., 2012), and as described below, hide in the endometrium of mares.

In healthy horses *S. zooepidemicus* is commonly found on mucus membranes of the upper respiratory tract and lower reproductive tract. However, *S. zooepidemicus* is also the most frequent cause of infectious endometritis in mares (Nielsen, 2005; Riddle et al., 2007; Nielsen et al., 2010; Overbeck et al., 2011), leading to sub- or infertility (Allen et al., 2007; Riddle et al., 2007; Petersen et al., 2015). Current available diagnostic tests for endometritis have limitations, and recently it has been demonstrated that the diagnostic sensitivity of culture-based techniques depend significantly on the compartment investigated e.g., a limited part the luminal endometrial surface using a swab; a large part of the luminal surface as with endometrial lavage or by including both the surface and deeper tissues as investigated using a biopsy (Nielsen, 2005; LeBlanc et al., 2007; Christoffersen et al., 2015). The endometrial lavage shows improved sensitivity for culturing bacteria compared to the swab, and is especially sensitive in diagnosing endometritis caused by *E. coli*, but there is a somewhat higher risk of contamination (LeBlanc et al., 2007; Leblanc and Causey, 2009), which has been lowed considerably after a double-guarded technique was introduced (Christoffersen et al., 2015). The biopsy is preferable over swabs as well, and has been used as a gold standard (Nielsen, 2005; Diel De Amorim et al., 2016). Whether a biopsy is more sensitive compared to lavage depends on the pathogen involved (Christoffersen et al., 2015). The biopsy can be advantageous when evaluating chronically infected mares, as these mares can carry *S. zooepidemicus* deep within the endometrium indicating that at least some strains of *S. zooepidemicus* seem to have the ability to enter and hide within the tissue for prolonged periods of time (Petersen et al., 2009; Rasmussen et al., 2013). This is further supported by clinical studies in infertile mares that were tested bacteriologically negative, despite extensive diagnostic efforts, yet were shown to carry a silent endometritis, when instilled with a bacterial growth medium that apparently can activate dormant streptococci (Petersen et al., 2015). It is however not clear where and how *S. zooepidemicus* specifically survives in the endometrium. Previous investigations have indicated that several other streptococcal species are able to invade host cells through different invasion mechanisms (Rohde and Chhatwal, 2013). Streptococcal invasins are most often surface exposed. The invasins promote uptake of the bacteria by the host through a triggering mechanism e.g., producing membrane ruffling (Dombek et al., 1999) or caveolae (Rohde et al., 2003). Some of the best described adhesins and invasins are the fibronectin binding proteins (FnBPs), among them the SfbI in *S. pyogenes*, which binds to fibronectin by inducing changes in its quaternary structure. The structural change promotes binding of fibronectin to host cell integrins and induction of intracellular signaling with cytoskeletal rearrangements and eventually internalization of the bacteria (Molinari et al., 2000; Ozeri et al., 2001). FnBPs have been identified in the *S. zooepidemicus* strains investigated in the current study indicating that *S. zooepidemicus* could use fibronecting binding proteins during cell invasion. Another important virulence factor in streptococci is the M protein, which is antiphagocytic but on the other hand stimulates opsonization by antibodies (Timoney et al., 1995). The M-protein is variable, primarily due to a hypervariable region, and has been used for typing purposes. Furthermore, M-proteins can act as invasins, but with varying internalization efficacy dependent on serotype (Rohde and Cleary, 2016). In *S. pyogenes* serotype M1 and M5 the hypervariable region, on the contrary to stimulating the immune system, seems involved in evading antibody attack through weak immunogenicity and antigenic variation (Lannergaard et al., 2011). *S. zooepidemicus* has a M-like protein (SzP), which is associated with virulence and opsonization as well (Hong-Jie et al., 2009), and has been explored as vaccine candidate (Velineni and Timoney, 2013; Lin et al., 2014), as well as other M-like proteins, the CspZ.1and CspZ.2 (Da Piedade et al., 2013; unpublished genome assembly of strain 1-4a). Like the FnBPs, M protein binding of fibronectin may lead to internalization and the M-like protein might thus have similar functions in *S. zooepidemicus*. We hypothesize that *S. zooepidemicus* can be located intracellularly and the aim of the current study was to investigate if *S. zooepidemicus* can invade and survive intracellularly in epithelial cells and at least partly explain why this bacterium can be difficult to diagnose and cause chronic and recurrent infections.

## Materials and methods

**Figure 1:**
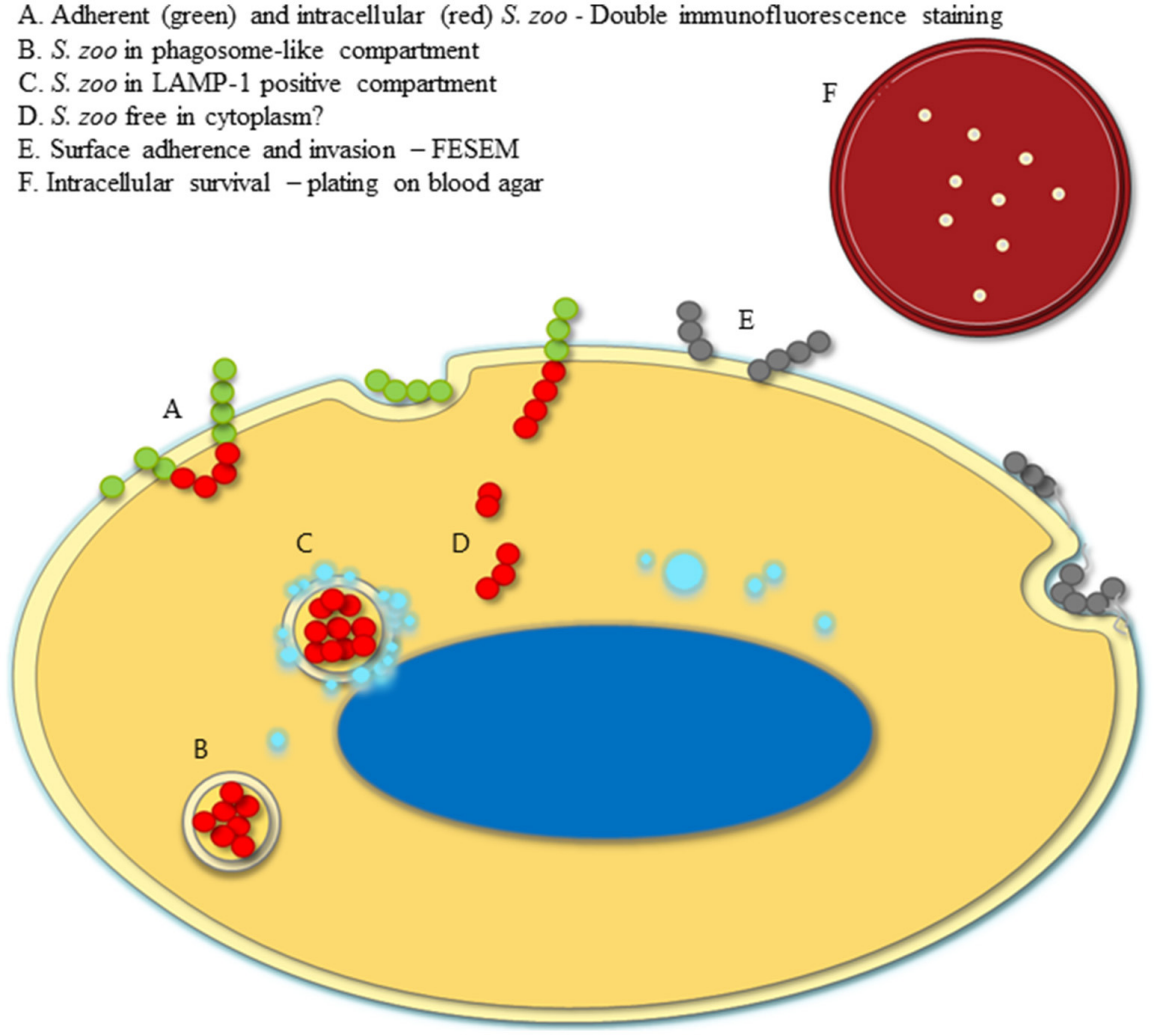
Illustration of experimental setup. A model of an epithelial cell infected with *S. zooepidemicus* is shown. The green and red circles represent double immunofluorescence stained *S. zooepidemicus*, where adherent extracellular bacteria are green, whereas intracellular bacteria are red **(A)**. Intracellular *S. zooepidemicus* are depicted in phagosome-like compartments. A majority of the compartments have no LAMP-1 signal **(B)**. However, some of the compartments are LAMP-1 positive (turquoise circles), meaning that lysosomes have fused with the compartment **(C)**. Few bacteria did not seem to be within a compartment, presented free in the cytoplasm **(D)**. Gray circles represent bacteria that were observed at the surface by scanning electron microscopy, and bacterial pili-like protrusions were expressed, illustrated with light gray lines **(E)**. Upper right corner a blood agar plate with colonies is shown to illustrate the growth and intracellular survival estimated by plating and retrieval of intracellular *S. zooepidemicus* after extracellular bacteria have been killed in the penicillin protection assay **(F)**.

A schematic illustration of the different approaches applied in this study is shown in Figure 1.

### Bacterial strains and growth conditions

To investigate if *S. zooepidemicus* was able to adhere to and invade epithelial cells *in vitro* two strains previously obtained from mares suffering from endometritis were selected. Strain S31A1 originates from the microbial culture collection at Department of Veterinary and Animal Sciences, University of Copenhagen, Denmark, whereas strain 1-4a was obtained from a research mare at the Maxwell H. Gluck Equine Research Center, University of Kentucky, Lexington, KY, USA. Stock cultures were stored at −80°C. For the infection assays, bacteria were streaked on blood agar (BA) plates, containing brain heart infusion (BHI) agar (BHI agar, CM 1136, Oxoid) added 5% bovine blood, and incubated overnight (ON) at 37°C. A single colony was transferred into BHI broth (BHI broth, CM 1135, Oxoid) and incubated ON at 37°C with shaking (125 rpm).

For control experiments, ON cultures of both strains were spun down, washed with PBS and heat-inactivated at 65°C in a heating block for 1 h in aliquots of 1 ml and plated on BA to document inactivation. The aliquots were stored at 4°C.

The non-invasive *Lactococcus lactis* subsp. *lactis* (strain IL1403; *L. lactis*) was included as a negative control to demonstrate that the internalization of the *S. zooepidemicus* was not due to a general passive uptake by the epithelial cells. From the −80°C stock *L. lactis* was streaked onto All Purpose Tween (APT) agar plate (APT agar, Merck) and incubated at 30°C for 2 days. Colonies were transferred into APT broth (APT broth, Difco, BD) and incubated ON at 30°C.

### Culture of human epithelial cell lines

The human epithelial cell lines HEp-2 (ATCC CCL 23) and HeLa (ATCC CCL 2) were used. Cells were maintained in modified Eagle's medium (DMEM, high-glucose, L-Glutamine, Phenol red, HEPES, Thermo Fisher Scientific) supplemented with 10% sterile filtered (Sartorius Minisart Filters 0.2 μm, Sigma-Aldrich) fetal bovine serum (FBS, Thermo Fisher Scientific) and 1% Penicillin/Streptomycin [Penicillin-Streptomycin (10,000 U/ml), Thermo Fisher Scientific], which will be referred to as Complete Medium in the following text. The cells were cultured in a humidified incubator with a 5% (v/v) CO_2_ atmosphere at 37°C in T-75 or T-150 cell culture flasks (Tissue Culture Flasks, TPP).

### Infection of HeLa and HEp-2

Cells 60–80% confluent were detached using trypsin (TrypLE Express Enzyme 1 X, no phenol red, Thermo Fisher Scientific), resuspended in pre-heated antibiotic free Complete Medium and seeded in 24-well tissue culture plates (Tissue Culture Test Plates, TPP) with an average of 4 × 10^4^ cells/well. For wells intended for imaging a glass coverslip (Coverslips Ø12 mm, 0.17 ± 0.01 mm thick, Menzel-Gläser, Thermo Fisher Scientific) was placed in the bottom of the well prior to seeding. 24-well plates were incubated 24 h to reach semi-confluence (65–80%) for optimal imaging. HEp-2 cells were used for the electron microscopy experiments and HeLa cells for the immunofluorescence and penicillin protection assays. Culture passages from 7–14 to 6–9 were used in the HeLa and HEp-2 cell experiments, respectively.

The bacterial inoculum was prepared from ON cultures, which had been adjusted to an optical density (OD) = 1 in PBS at 600 nm and immediately prior to inoculation diluted in pre-heated antibiotic free Complete Medium 1:800 for live bacteria, and 1:400 for the inactivated *S. zooepidemicus*. Each well was inoculated with 1 ml bacterial suspension, except the negative control, which was inoculated with media without bacteria. An aliquot of each inoculum was plated to enumerate the colony forming units/ml (CFU/ml). The average inoculum for strain 1-4a was (3.9 ± 1.6) × 10^5^ and for strain S31A1 (2.2 ± 0.4) × 10^6^ CFU/ml (mean ± SD), giving the multiplicity of infection (MOI) of 1:10 and 1:55, respectively. The entire length of the co-culturing was 23 h. Samples were taken at various time point during the experiment.

### Quantitative analysis of adherent and intracellular *S. zooepidemicus*

To quantify adherent and intracellular bacteria double immunofluorescence staining was performed at different time points of infection, as previously described (Molinari and Chhatwal, 1998) with some modifications. Coverslips were washed in PBS and fixed in 24-well plates containing 1% formaldehyde in a modified HEPES buffer (0.1 M HEPES, 0.01 M calciumchlorid dihydrat, 0.01 M magnesiumchlorid hexadydrat, 0.09 M saccharose) adjusted to pH 6.9. Three independent experiments were performed in triplicate. The time points for fixation were 1 h 45 min, 2 h 45 min, and 3 h 30 min, respectively. Fixed coverslips were kept at 4°C until staining. Coverslips were washed once in wells with PBS containing 0.35 M Glycine for 20 min, and twice with PBS alone. To stain extracellular adherent bacteria samples were incubated with a 1:200 dilution of rabbit anti-Streptococcus Group C antibody (Pineda Antikörper-Service, Berlin) in PBS for 1 h. The samples were washed in PBS and incubated for 30 min with a 1:200 dilution of AlexaFluor 555 (AF 555) conjugated goat anti-rabbit IgG antiserum (ThermoFisher Scientific) in PBS. Subsequently, the samples were washed in PBS and epithelial cells were permeabilized with 0.1% Triton X-100 (Sigma-Aldrich) in PBS for 40 min. washed again in PBS and incubated with a 1:200 dilution of the rabbit anti-Streptococcus Group C antiserum for 1 h, washed in PBS, and incubated with a 1:200 dilution of AlexaFluor 647 (AF 647) conjugated goat anti-rabbit IgG antiserum (ThermoFisher Scientific) in PBS for 30 min to stain all *S. zooepidemicus*, extracellular adherent, and intracellular bacteria. To visualize DNA the samples were washed again and stained with 0.5 μg/ml 4′,6-diamidino-2-phenylindole (DAPI) (Sigma-Aldrich) in PBS for 10 min (Figure 1). After final washing, the coverslips were mounted on microscope slides with mounting media (ProLong® Gold antifade reagent, ThermoFisher Scientific). The following day edges were sealed with nail polish.

The rabbit anti-Streptococcus Group C antiserum had been purified with a protein A column (Protein A Sepharose CL-4B, GE Healthcare Life Sciences) by affinity chromatography.

All tubes containing diluted antibodies were coated with 10% FBS to avoid antibodies binding to the plastic. The antibodies were mixed as one batch for the staining of all samples to minimize variation in fluorescence intensities. Each washing step included washing in two 250 ml beakers with PBS. PBS was changed between each washing step.

Images were acquired with a fluorescent microscope (Zeiss Axio Scan.Z1). Zeiss filter nr. 01, 38HE, 43HE, and 50 were used and a LED light source was applied. Larger areas were scanned and stitched to one image covering larger parts of the coverslip resulting in an average of 11.500 HeLa cells examined per time point for the three independent experiments in total, and about half for the inactivated samples, as these were only present as duplicates. Images were acquired as Z-stacks and post processed using the extended depth of focus function in the software [Zeiss ZEN 2 (blue edition)]. A generalized image recording setup was applied to all slides, with only the exposure time for the AF 647 varying slightly between the two strains.

The quantitative analysis of the adherent and intracellular *S. zooepidemicus* was performed using the Zeiss ZEN 2—(blue edition) software. The algorithm processed the fluorescent intensities, and calculated an area based on thresholds set for each channel. Proper setting of thresholds, together with setting a minimum size for objects, discarded most unwanted/unspecific signal. The thresholds for the double stained adherent *S. zooepidemicus* were aligned to provide the same sized area for both stains (AF 555 and AF 647). The DAPI signal threshold was set to delineate the HeLa cell nucleus, enabling estimation of the total number of cells per image analyzed. The green fluorescent channel was used to visualize autofluorescence often located in green fluorescence spectrum when cells are fixed with formaldehyde. The HeLa cell nucleus and cytoplasm showed autofluorescence and could thus be outlined from the background and the threshold was set to delineate the HeLa cells as clearly as possible and hereby excluding areas not covered by cells from the analysis. The bacterial signal was set to be dependent on the autofluorescence, meaning that primarily bacteria in contact with cells would be included in the analysis. For strain 1-4a one global profile was used to analyze all slides, except for the autofluorescence threshold which was fitted for each sample, as it varied. For strain S31A1 one global setting did not result in a good alignment of the two signals for all samples. The thresholds were thus fitted to give the best alignment for each sample. All analyzed areas were proof-read to discard signals from dead HeLa cells and signals from unspecific binding. As controls inactivated *S. zooepidemicus* and *L. lactis* samples, fixed at 3 h 30 min, were included and imaged under similar conditions. All analyses and proof-reading were done blinded.

Equation for calculating the fluorescent adherent signal per HeLa cell:

$$\begin{array}{l} Adherent\;bacterial\;signal=\;\frac{Adherent\;bac.\;signal\left(\mu m2\right)\;}{Number\;of\;HeLa\;cells} \end{array}$$

Equation for calculating the fluorescent intracellular signal per HeLa cell:

$$\begin{array}{l} Intracellular\;bacterial\;signal\\ =\;\frac{\left(Total\;bac.\;signal-Adherent\;bac.\;signal\right)\mu m2}{Number\;of\;HeLa\;cells} \end{array}$$

### Association of intracellular *S. zooepidemicus* with lysosomal markers

To investigate if the internalized *S. zooepidemicus* cells were located in phagosomal compartments which fused with late endosomal-lysosomal compartments, we used primary antibodies against human lysosomal-associated membrane protein (LAMP-1, Purified Mouse Anti-Human CD107a, Clone H4A3, BD Pharmingen) one of the marker enzymes of lysosomes (Figure 1). For these studies coverslips were washed and fixed in 24-well plates in 1% formaldehyde as described above. Three independent experiments were performed in duplicate. Coverslips were fixed at time points prior to the treatment with penicillin, (2 h 45 min and 3 h 30 min) and after treatment with penicillin 2 μg/ml (Penicillin G sodium salt, Sigma-Aldrich; 11 and 23 h).

The staining protocol was similar to the one describe above, but included two extra steps. After the permeabilization with Triton X-100, samples were incubated with a 1:50 dilution of primary antibodies LAMP-1 in PBS for 1 h, washed and incubated with a 1:200 dilution of AF 647 conjugated goat anti-mouse IgG antiserum (ThermoFisher Scientific) in PBS for 30 min, and the protocol proceeded to the end. The first bacterial staining was with AlexaFluor 488 (AF 488) conjugated goat anti-rabbit IgG antiserum (ThermoFisher Scientific) and the staining of bacteria after permabilization was with AF 555 conjugated goat anti-rabbit IgG antiserum (ThermoFisher Scientific). The samples were examined using an upright laser scanning confocal microscope (Zeiss LSM 710). Main beam splitter (MBS) 488/561/633 and MBS 405 where applied. Master gain and laser intensities were adjusted for each image acquisition to optimize image quality. The image acquisition was carried out using the ZEN 2 (black edition) confocal scanning software. The negative controls (NC) were HeLa cells fixed at 23 h and subjected to the LAMP staining protocol.

### Field emission scanning electron microscopy (FESEM)

For visualization of the *S. zooepidemicus* adhesion and invasion of epithelial cells, infected semi-confluent HEp-2 cells grown on coverslips in antibiotic free Complete Media, as previously described, were fixed at 1 h 30 min and 3 h 30 min with 5% formaldehyde and 2% glutardialdehyde in modified HEPES buffer for at least 1 h at 4°C, then washed three times with modified HEPES buffer. Samples were then dehydrated with a graded series of acetone (10, 30, 50, 70, 90, 100%) on ice for 15 min for each step. Samples in the 100% acetone step were allowed to reach room temperature before another change of 100% acetone. Samples were then subjected to critical-point drying with liquid CO_2_ (CPD300 Auto, Leica, Wetzlar). The dried samples were coated with a gold palladium film by sputter coating (Bal-Tec SCD 500, Balzers, Liechtenstein) before examination in a field emission scanning electron microscope (Zeiss Merlin) using the Everhart Thornley SE-detector and the InLens SE-detector in a 75: 25 ratio at an acceleration voltage of 5 kV (Figure 1). As controls both inactivated *S. zooepidemicus* and *L. lactis* samples were included in the study, fixed at 3 h 30 min and imaged under similar conditions. Two independent experiments were performed in duplicate.

### Growth and adhesion/invasion

To estimate the total growth, adherence and invasion *S. zooepidemicus* were enumerated by plating appropriate dilutions on BA plates at the time of inoculation, and after 2 h and 3 h 30 min of co-culturing with HeLa cells in the antibiotic free Complete Media. CFU was estimated from two fractions. First the Complete Media was taken out of the well, and the well was washed twice with 1 ml PBS, which was added to the media already taken from the well. This fraction was plated in appropriate dilutions on BA to enumerate bacterial cells in the media. The well was further processed to enumerate the adherent and intracellular fraction by adding 250 μl TrypLE express and incubate for 3–5 min and then the HeLa cells were lysed by adding 750 μl Triton X-100 0.2% and appropriate dilutions were plated on BA. Three independent experiments were performed in duplicate.

Equation for calculating the relative ratio of strain 1-4a being adherent and intracellular compared to the strain S31A1 being adherent and intracellular:

$$\begin{array}{l} Relative\;Adherence\;and\;Invasion\\ =\;\frac{1-4a\;CFU\;adh.+inva./1-4a\;CFU\;total\;}{S31A1\;CFU\;adh.\;+inva./S31A1\;CFU\;total} \end{array}$$

### Penicillin protection assay (intracellular survival)

To investigate to which extend the internalized *S. zooepidemicus* survived intracellularly we used a Penicillin Protection Assay, exchanging the Complete Media without antibiotics with a pre-heated Complete Media containing 2 μg/ml penicillin at 2 h post-infection, to kill extracellular *S. zooepidemicus*. The wells were washed once with pre-heated PBS in between media change. The adherent and intracellular bacteria were determined by plating immediately prior to addition of penicillin. The wells were then further incubated. At 2, 9, and 21 h after penicillin was addedand appropriate dilutions plated on BA (Figure 1). Three independent experiments were performed in duplicate.

Equation for calculating the relative ratio of the intracellular survival of strain 1-4a compared to the intracellular survival of strain S31A1:

$$\begin{array}{l} Relative\;Survival=\\ \frac{1-4a\;CFU\;survival/1-4a\;CFU\;adh.+inva.\;\left(2\;h\right)\;}{S31A1\;CFU\;survival/S31A1\;CFU\;adh.+inva.\;\left(2\;h\right)} \end{array}$$

### Statistical analysis

Data were analyzed performing two-way ANOVA followed by Tukey's multiple comparison test for the double immunofluorescence data where three independent experiments were performed in triplicate, and by performing two-way ANOVA followed by Sidak's multiple comparison test for the intracellular survival data, where the strain S31A1 was mathematically adjusted to have the same inoculum size (MOI of 1:10) as strain 1-4a, to enable statistical comparison of the strains. The penicillin protection assay was performed as three independent experiments in duplicate. Data was analyzed using GraphPad Prism version 7.00 for Windows, GraphPad Software, La Jolla California USA, www.graphpad.com and expressed as means with standard deviation. *P*-values < 0.05 were considered significant.

## Results

### *S. zooepidemicus* adhered and invaded HEp-2 cells through different invasion mechanisms and formed pili-like appendages

**Figure 2:**
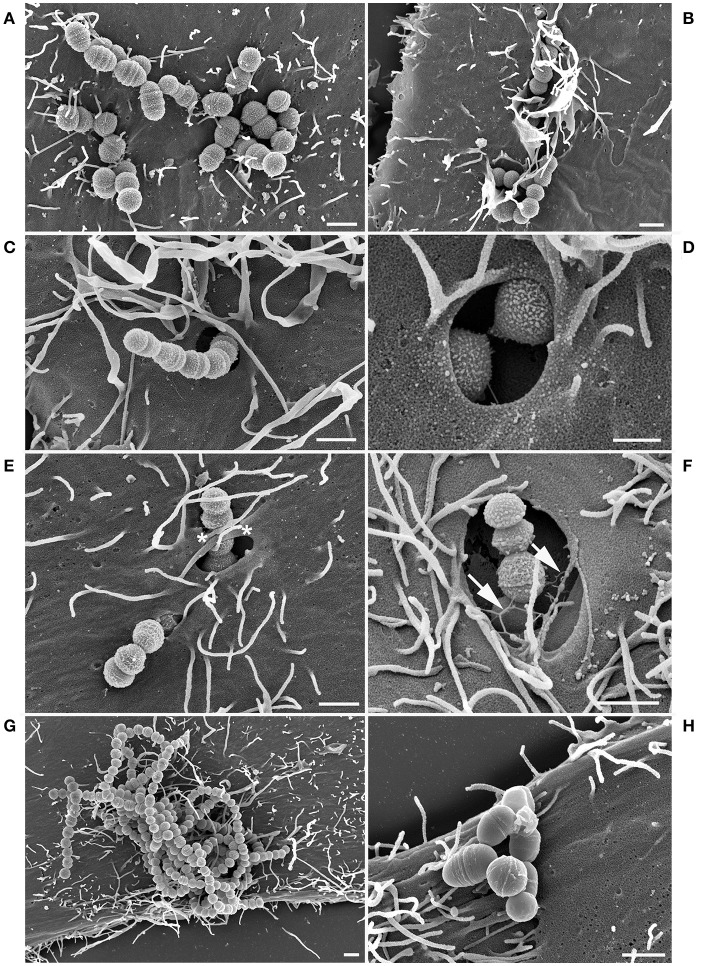
FESEM analysis of HEp-2 cells co-cultured with *S. zooepidemicus* strain 1-4a for 1.5 h and 3.5 h. Adherent bacteria **(A)** trigger three different types of invasion. Membrane ruffling and cytoskeletal rearrangements **(B)**, large invaginations in the plasma membrane of the HEp-2 cells **(C–D)**, and engulfment from the middle of the chain causing the cell membrane to form protrusions which overgrow the bacterial chain (^*^) **(E)** were observed after 1.5 h of co-culturing. At the later time point (3.5 h) the bacterial cell surface showed expression of pili-like appendages (arrows) **(F)**. Heat-inactivated strain 1-4a was adherent but showed no invasion **(G)** as the non-invasive control *L. lactis* **(H)** after 3.5 h of co-culturing. Bars represent 1 μm in **(A–C)** and **(E–H)** and 0.5 μm in **(D)**.

**Figure 3:**
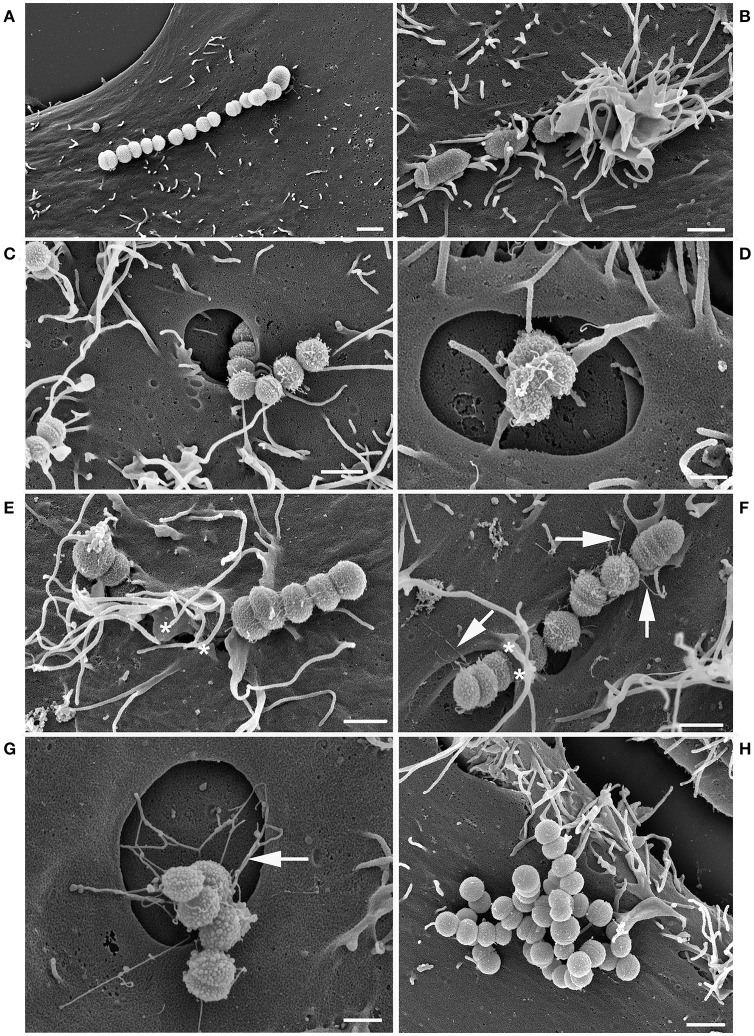
FESEM analysis of HEp-2 cells co-cultured with *S. zooepidemicus* strain S31A1 for 1.5 and 3.5 h. Adherent bacteria **(A)** trigger three different types of invasion. Membrane ruffling **(B)**, large invaginations in the plasma membrane of the HEp-2 cells **(C,D)**, and engulfment from the middle of the chain causing the cell membrane to form protrusions which overgrow the bacterial chain(^*^) **(E)** were observed after 1.5 h of co-culturing. Pili-like appendages can also be found (arrows) **(F)**. The pili-like appendages were seen in connection to the large invaginations as well after 3.5 h **(G)**. Heat-inactivated strain S31A1 adherent, but non-invasive after 3.5 h **(H)**. Bars represent 1 μm in **(A–C)** and E-H and 0.5 μm in **(D)**.

The adhesion and invasion of the *S. zooepidemicus* strains to HEp-2 cells were investigated using field emission scanning electron microscopy (FESEM). *S. zooepidemicus* adhered to the cell (Figures 2A, 3A) and three morphologically different invasion mechanisms for both strains were visualized; (a) triggering cytoskeletal rearrangements, i.e., membrane ruffling (Figures 2B, 3B), (b) formation of large invaginations in the HEp-2 cell membrane which serve as invading portal for the bacteria (Figures 2C,D, 3C,D), and (c) invading with bacterial engulfment taking place in the middle part of an adherent streptococcal chain, with the host cell membrane forming protrusions overarching the bacterial chain and thereby internalizing *S. zooepidemicus* (Figures 2E, 3E). During the internalization process *S. zooepidemicus* showed changes in phenotype. The bacterial cell wall became less smooth and formation of pili-like appendages from the cell wall which connected to other bacteria or to the host cell surface were seen (Figures 2F, 3F,G). Strain S31A1 appeared to express more of such appendages compared to strain 1-4a. In general appendages were more prominent at the later time points of infection. The FESEM analysis indicated that strain 1-4a was more adhesive and invasive compared to strain S31A1.

Heat-inactivated *S. zooepidemicus* were observed adhering to the HEp-2 cells but were generally not invading the cells, supporting that the internalization was an active process promoted by the interaction between the pathogen and the host cell (Figures 2G, 3H). For the heat-inactivated strain 1-4a some engulfment by HEp-2 cells were seen at very low frequencies. The control strain *L. lactis* showed adherence, but no internalization was detectable (Figure 2H).

### Quantitative analysis of the adherent and intracellular *S. zooepidemicus*

To analyze the adhesion and invasion efficiencies over time and between the two strains, bacteria were co-cultured with HeLa cells. At three different time points the bacteria were double immunofluorescence stained to differentiate extra- and intracellular bacteria, and analyzed using an algorithm based on the fluorescent intensities.

**Figure 4:**
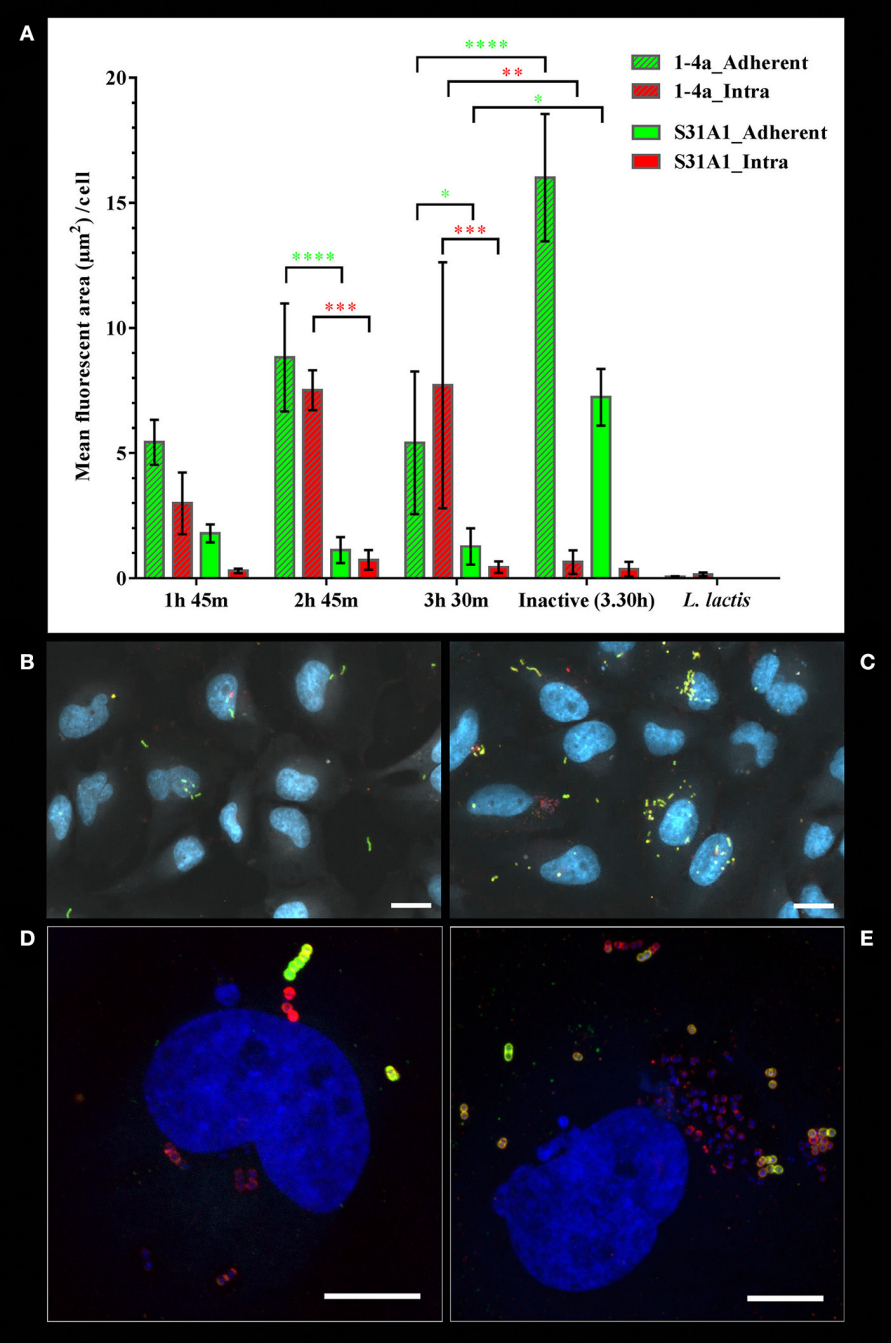
Adherent and intracellular *S. zooepidemicus* determined by double immunofluorescence staining and quantitative analysis of fluorescent intensities. The *S. zooepidemicus* strains were co-cultured with HeLa cells. The infection was stopped at various time points (1 h 45 m, 2 h 45 m, and 3 h 30 m) immunofluorescence stained and analyzed as described in Materials and Methods. **(A)** The graph shows bacterial fluorescent cell area per HeLa cell, were the extracellular/adherent signals are shown with green bars and the intracellular signals shown with red bars. Strain 1-4a bars are slashed, while strain S31A1 bars have no filling. Means ± SD from three independent experiments are shown. Green asterisks mark significant difference between extracellular/adherent bacterial signals, and red asterisk mark significant difference between intracellular bacterial signals. ^*^p < 0.05, ^**^p < 0.01, ^***^p < 0.001, ^****^p < 0.0001. **(B)** Overview image showing adherent (green and yellow) and intracellular (red) bacteria for strain S31A1. **(C)** Overview image showing adherent and intracellular bacteria for strain 1-4a. For **(B)** and **(C)** autofluorescence, demarking the HeLa cells, is shown in white, and for **(B–E)** DNA is shown in blue. **(D)** Maxprojection of a confocal stack showing a strain S31A1 infected cell **(E)** Maxprojection of a confocal stack showing a cell highly infected with strain 1-4a. **(B,C)** Axioscan images, scale bars are 20 μm. **(D,E)** confocal images, scale bars are 10 μm.

The analysis of strain 1-4a showed increase in intracellular bacteria from the early time point to the later, meaning that invasion was a process which progressed with time (Figure 4A). At the later time point more variations could be seen, primarily due to one of the three studies diverging from the other two. The adherence of strain 1-4a was highest at 2 h 45 min, where the bacterial-cell interaction seemed to have reached saturation, as most cells were infected. Strain S31A1 adhered and invaded significantly less compared to strain 1-4a at 2 h 45 min and 3 h 30 min, and a larger fraction of S31A1 remained extracellular (Figure 4A). Observations from the fluorescence microscope images showed strain S31A1 with less both adherent and intracellular bacterial cells per HeLa cell (Figure 4B) compared to strain 1-4a (Figure 4C). Figures 4D,E illustrate the same observations shown in detail with a single infected HeLa cell. Representative images of the three independent experiments are shown. None of the strains presented an even distribution of bacteria per HeLa cell, as some HeLa cells were heavily infected, while others had no or very few adherent and intracellular bacteria (Figures 4B,C). Identical observations were made in the FESEM analysis of the infected HEp-2 cells (Figures 2, 3). In a pilot study we did double immunofluorescence staining on infected HEp-2 cells as well, and got similar results, although quantification was not performed (data not shown).

The adherent bacterial signal form the control experiments with the heat-inactivated *S. zooepidemicus* was significantly higher than the adherent bacterial signal from live bacteria. This artifact occurred as the inactivated bacteria tended to clump giving an intense fluorescent signal, as well as sticking to the coverslip at areas were cells were not present to a larger extent than observed for the live bacteria. Importantly the intracellular signal was very low, indicating some background fluorescence and a very low passive uptake of inactivated *S. zooepidemicus* (Figure 4A). The low signal from *L. lactis* demonstrated the overall background fluorescence in the assays, with the thresholds used, and confirmed the specificity of the primary antibodies, which should only bind to Group C streptococci (Figure 4A).

The quantitative analysis can be very sensitive, but at the cost of less specificity giving problems of discriminating low invasive strains like S31A1 from the inactivated samples. To be conservative the analysis should be regarded as an approximation, but a reasonable one, as the other approaches used in this study showed similar results.

### Growth and intracellular survival

The start inoculum for the two strains were different even though their ODs had been adjusted in a similar manner, giving a start MOI for strain 1-4a of 1:10 and for strain S31A1 1:55, corresponding to an average of 10 or 55 *S. zooepidemicus* CFU per HeLa cell, respectively. After 2 h of co-culture both strains had multiplied. Strain 1-4a had multiplied 8.8 times, while S31A1 had multiplied 12.2 times. The strain differences in adherence and invasion efficiencies was evident in this assay as well, where 1-4a was found 18 times as likely to be adherent or intracellular at 2 h post infection compared to strain S31A1. The number of adherent and intracellular bacteria reduced over time for both strains, indicating no intracellular growth (data not shown).

To investigate if the internalized *S. zooepidemicus* survived intracellularly, penicillin protection assays were performed. The intracellular survival was significantly different between the strains 2 h post-penicillin addition. Four percent of strain 1-4a survived, which made 1-4a 6.3 times as likely to survive compared to strain S31A1. This was expected, as the intracellular fraction of strain 1-4a was higher, as determined by the quantitative analysis. The intracellular survival decreased significantly for both strains and reached similar levels at 9 and 21 h post-penicillin addition (Table 1).

**Table 1:** Intracellular survival.

**Hours penicillin treatment**	**Intracellular survival, % ± SD**		**Relative Survival**
	**1-4a**	**S31A1**	
2 h^*^	4.02 ± 1.96	0.69 ± 0.20	6.3
9 h	0.24 ± 0.05	0.16 ± 0.02	1.5
21 h	0.03 ± 0.02	0.04 ± 0.01	0.8

*The S. zooepidemicus strains were co-cultured with HeLa cells. At 2 h post-infection cells were washed and media containing penicillin was added. The number of surviving bacteria was determined by plating 2, 9, and 21 h after penicillin had been added. Results were expressed as percentage survival (% recovered CFU divided by CFU at 0 h when penicillin was added), as described in Material and Methods. Means ± SD of three independent experiments are shown*.

* *Significant difference between strains (p < 0.05). Relative Survival when the strains were compared (equation in Material and Methods)*.

**Figure 5:**
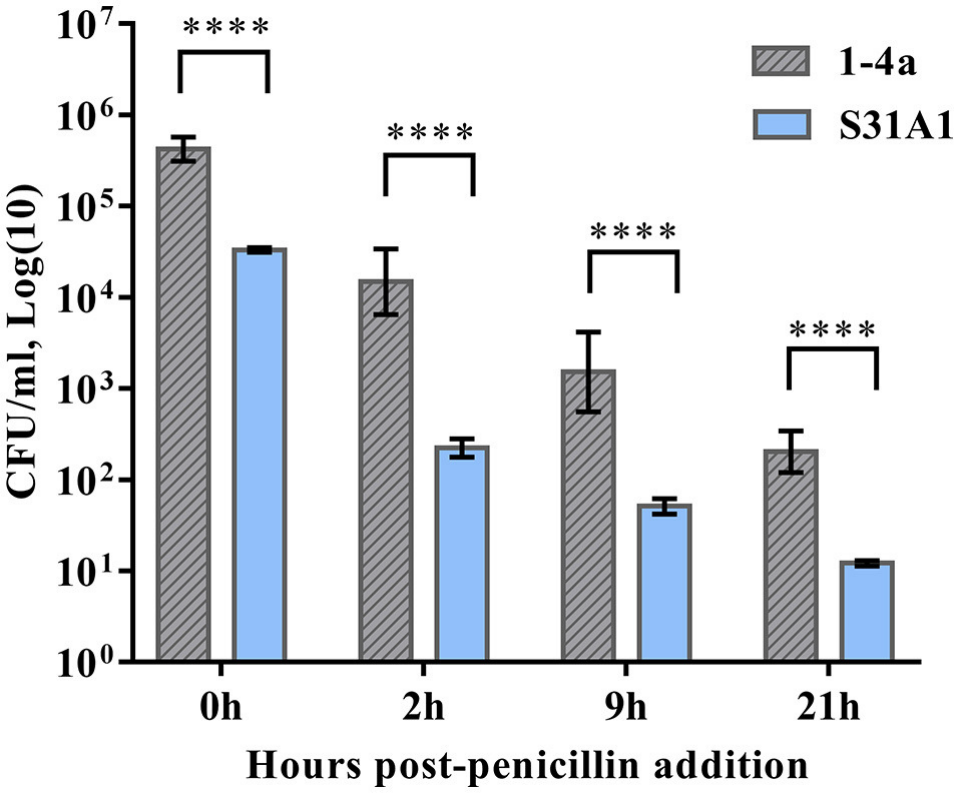
The *S. zooepidemicus* strains were co-cultured with HeLa cells. At 2h post-infection the cells were washed and Complete Media containing penicillin was added. The number of surviving bacteria was determined by plating 0, 2, 9, and 21 h post-penicillin addition. Results were expressed as Log(10) CFU/ml. Means ± SD of three independent experiments are shown. ^****^p < 0.0001.

Expressing the survival as CFU/ml instead of as percentages however reveals a highly significant difference between the strains in the actual number of surviving bacteria at 2, 9, and 21 h post-penicillin addition (*P* < 0.0001), which again can be explained as a significant strain dependent difference in invasiveness (Figure 5). However, the results indicate that the bacteria that do invade have a similar chance of survival, as the percentage of survival when comparing the two strains was similar at 9 and 21 h post-penicillin addition (Table 1). The strain S31A1 was mathematically adjusted to have the same inoculum size (MOI of 1:10) as strain 1-4a, to be able to compare the strains statistically.

### Intracellular trafficking

**Figure 6:**
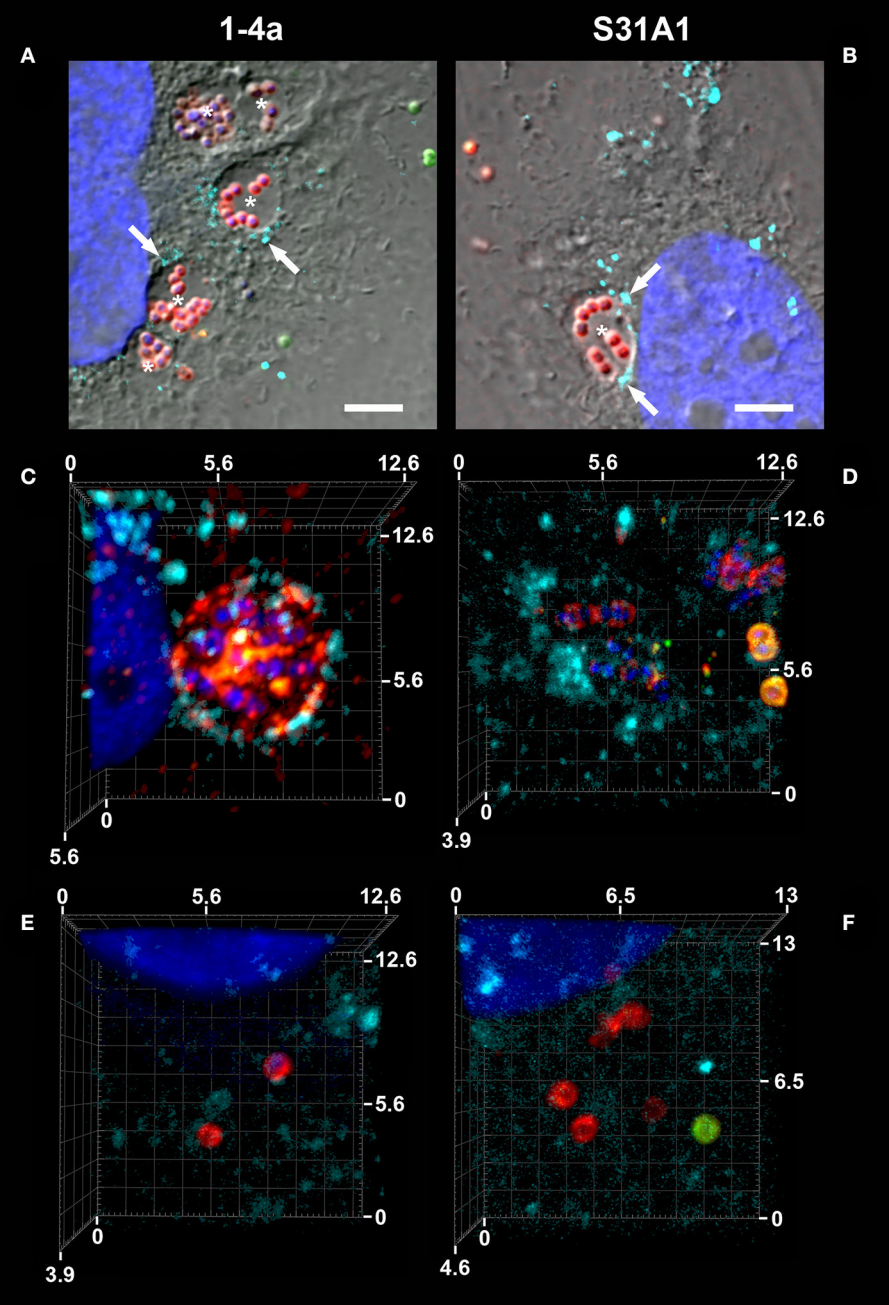
Intracellular trafficking and staining for human lysosomal-associated membrane protein, LAMP-1. The *S. zooepidemicus* strains were co-cultured with HeLa cells and samples from different time points were immunofluorescence stained for LAMP-1 (turquoise), and the bacteria were double immunofluorescence stained (intracellular red; extracellular/adherent green), DNA is shown in blue. **(A)** Strain 1-4a in phagosome-like compartments (marked with asterisks ^*^), with varying degrees of LAMP-1 association (arrows), 11 h post-infection. **(B)** Strain S31A1 in phagosome-like compartments (marked with asterisks ^*^), with some LAMP-1 association (arrows), 3 h 30 min. **(C)** Strain 1-4a in a phagolysosome-like compartment, 3 h 30 min. **(D)** Strain S31A1 in a phagolysosome-like compartment, 3 h 30 min. **(E)** Intracellular strain 1-4a with no clear LAMP-1 association, 23 h. **(F)** Intracellular strain S31A1 with no clear LAMP-1 association, 23 h. All images are recorded with a confocal microscope. **(A,B)** with a DIC overlay, scale bars are 10 μm. **(C–F)** 3D images created from Z-stacks in Zen 2 (blue edition), scale bars are in μm.

The double immunofluorescence images indicated that the majority of the internalized *S. zooepidemicus* were contained in phagosome-like compartments as bacteria were densely packed inside the host cells. For strain 1-4a these phagosome-like structures contained large numbers of bacteria, often more than 10 bacteria were observed (Figure 6A), when compared to strain S31A1, in which these phagosome-like compartments generally contained <10 bacteria (Figure 6B).

To evaluate if invaded bacteria follow the classical endocytic pathway with subsequent fusion with lysosomes, we investigated if lysosomes would fuse with the phagosome-like compartments at later time points of infection. For this purpose fluorescence staining of LAMP-1 of the lysosomes in HeLa cells was performed. In general LAMP-1 association with the intracellular *S. zooepidemicus* was not a frequent finding, but so called phagolysosomes could be found for both strains demonstrating that some of the invading *S. zooepidemicus* traffic via the classical endocytic pathway in the host cell (Figures 6C,D). Examples of LAMP-1 negative compartments containing bacteria were found, as well as were intracellular bacteria where no clearly defined compartment could be observed. These could represent bacteria residing in membrane-surrounded compartments different to phagolysosomes or bacteria roaming free in the bacterial cytoplasm (Figures 6E,F). In a pilot study we stained for LAMP-1 in infected HEp-2 cells, and found *S. zooepidemicus* in phagolysosomes more frequently, although quantification was not done (data not shown).

## Discussion

By applying fluorescence and electron microscopic approaches we showed that *S. zooepidemicus* was able to adhere to and invade non-professional phagocytic human epithelial cell lines in a cell culture model.

### Invasion mechanisms and pili-like appendages

Three morphologically different invasion mechanisms were demonstrated in this study (Figures 2, 3). All three mechanisms have previously been described for other streptococcal species like *S*. *pyogenes, S. suis*, and *S. dysgalactiae*, although not all three different mechanisms in one single strain (Calvinho and Oliver, 1998; Molinari et al., 2000; Benga et al., 2004; Rohde et al., 2012). The membrane ruffling of the epithelial cell membrane has been termed a “trigger mechanism” which promotes cytoskeletal rearrangements and accumulation of actin (Dombek et al., 1999; Molinari et al., 2000). The bacteria are taken up in large vacuoles, which usually will follow the classical endocytic pathway with fusion with late endosomes or phagolysosomes (Cossart and Helenius, 2014). Previous studies have indicated that the M protein is triggering this uptake mechanism, and in *S. pyogenes* serotype M1, the M protein acts as a primary invasin (Cue et al., 1998). M-like proteins have been found in *S. zooepidemicus* and we have identified M-like proteins in the strains investigated in this study as well (Da Piedade et al., 2013; unpublished genome assemply of strain 1-4a). These proteins could be involved in the triggering of membrane ruffling, but further specific studies would be needed to confirm this hypothesis. The engulfment from the middle of the streptococcal chain also promotes cytoskeletal rearrangements, but with the formation of cellular membrane protrusions, that fuse over the middle part of the bacterial chain, leading to engulfment as previously described for *S. dysgalactiae* subsp. *equisimilis* (Rohde et al., 2011) and *S. pyogenes* (Rohde and Cleary, 2016). The molecular background for this uptake mechanism has to our knowledge not been clarified. For *S. pyogenes*, triggering of cell cytoskeleton rearrangements and the subsequent invasion typically leds to fusion with lysosomes forming phagolysosomes intracellularly which, in most cases, will result in degradation of the bacteria (Rohde and Cleary, 2016). The primary uptake of the strains investigated in the current study was through large invaginations in the epithelial cell membrane, which in previous studies have been linked to the co-opting of host cell caveolae (Molinari et al., 2000; Rohde et al., 2003; Parton and Simons, 2007). A non-classical endocytic pathway with caveolae maturing to membrane-bound compartments called caveosomes has been investigated extensively in the early 2000's. The intriguing observation about this special pathway was that the caveosome would not fuse with lysosomes, hereby retaining a neutral pH, and the pathogen contained in the caveosome would not be degraded (Pelkmans and Helenius, 2002). However, one of the groups presenting the theory have been debating recently whether this specific caveosome pathway exists, and now argue that caveolae are indeed part of an endocytic pathway and will be directed for intracellular degradation (Parton and Howes, 2010). Our results suggested caveolae-like uptake as the main mode of entry, but also indicated limited fusion with lysosomes. It can be discussed if some of the large compartments seen in our study actually had fused with lysosomes, as a LAMP-1 signal could be detected in close proximity to the compartments, but not necessarily surrounding the entire compartment (Figures 6A,B). For future immunofluorescence studies it would be relevant to stain for both (a) caveoline-1(Asmat et al., 2014), to investigate if actually a caveolae mediated uptake is taking place, and (b) a fluorescent dye that stains acidic compartments in live cells to investigate if the intracellular compartments containing *S. zooepidemicus* have been acidified by fusion with lysosomes (Benga et al., 2004).

The mechanism of uptake into host cell caveolae has been linked to the binding of fibronectin to the streptococcal FnBPs and the subsequent binding to the host cell α5β1 integrins leading to integrin clustering and caveolae aggregation, which results in the forming of the large invaginations in the host cell membrane without accumulation of actin (Rohde et al., 2003). It can thus be speculated that the FnBPs found in the *S. zooepidemicus* strains investigated in this study could trigger this uptake mechanism (Da Piedade et al., 2013; unpublished genome assemply of strain 1-4a), even though differences in the sequence of the FnBPs between streptococcal species can lead to different uptake mechanisms (Rohde et al., 2011).

The pili-like appendages expressed by both strains studied (Figures 2F, 3F,G) could serve as adhesins anchoring the bacteria more tightly to the host cell after the initial adhesion has taken place. Pili have been described for other streptococcal species and Gram-positive bacteria in general, with adhesins located at the tip of the pili (Ton-That and Schneewind, 2004; Rosini et al., 2006; Scott and Zähner, 2006; Fälker et al., 2008; Melville and Craig, 2013). Putative pili loci have been identified in *S. zooepidemicus* (FimI-III) and loci encoding sortases (Holden et al., 2009), which are essential for the assembly of the pili (Ton-That and Schneewind, 2004). Our strain S31A1 has four sortase C genes (SrtC 1-4) and the sortase A gene (SrtA), six fimbrial subunit proteins (FszA-F), a putative pilus domain with high identity to the *S. zooepidemicus* SzH70 (SZO_08560), and a gene identical to the RofA/Nra-like fimbrial gene transcriptional regulator (sez_1823) in *S. zooepidemicus* strain MGCS 10565 (Beres et al., 2008; Ma et al., 2011; Da Piedade et al., 2013). In strain 1-4a we found that a large part of this gene cluster has been disrupted by a transposon and integrase, resulting in the loss of SrtC 2-4, FszC-F, and the RofA/Nra-like genes. This might explain the lower pili expression in strain 1-4a observed in the FESEM images. Besides acting as adhesins, the pili-like appendages appear to facilitate bacterial co-aggregation and could be part of early biofilm formation (Nobbs et al., 2009). Biofilm formation has previously been described in *S. zooepidemicus* (Yi et al., 2013, 2016). In our study pili were also expressed on *S. zooepidemicus* in the process of being internalized, and it would be interesting to further characterize these structures and their role in the adhesion and invasion process as this may be a critical component in the host-pathogen interaction.

### Quantitative analysis of the adherent and intracellular *S. zooepidemicus*

The double immunofluorescence staining technique applied in this study was used to discriminate between adherent extracellular and intracellular bacteria and allowed us to assess the internalization kinetics on both a qualitative and quantitative basis, which is an advantage to standard CFU counting techniques (Dziwisch et al., 1988). The semi-automatic algorithm allowed us to collect and analyze data from a larger number of samples and in a more objective manner, than would have been possible from manual counting, and confirmed the strain differences demonstrated by the other techniques applied. Further advantages of the fluorescent microscopy approach was the DAPI staining of the DNA, which made it possible to access cell viability, as cells that go into apoptosis will exhibit disrupted nuclei with loss of integrity. The analysis could thus be adjusted to only report data from cells that appeared viable, and the bacterial signal could be normalized to the number for HeLa cells present.

However, the algorithm had certain limitations. It was a challenge fitting thresholds, as the adhesion and internalization process were evolving at different stages in different cells, and it was not possible to get a perfect fit for every situation. The aim was thus to get the best fit, while being blinded to minimize subjective selections. The ideal situation would be if one global setting could be applied to all samples, but unfortunately that would result in a misleading interpretation of the images.

Strain 1-4a showed more variation than strain S31A1 (Figure 4A), which may arise from the strong invasiveness by 1-4a in some of the HeLa cells resulting in cell damage and apoptosis due to large bacterial loads. The strain variation as a consequence of cellular damage is not an unusual finding, and has been reported for other streptococci (Rohde et al., 2003; Benga et al., 2004; Kaplan et al., 2006).

### Intracellular survival and persistence

*S. zooepidemicus in vitro* adherence and invasion to eukaryotic cells has been reported previously using the standard antibiotic protection assays for the two pig isolates, American Type strain ATCC35246 and Chinese strain C55138 (Wei et al., 2013; Xu et al., 2016a,b). The adhesion levels were comparable to the strains of the current investigation, and the invasion efficiencies comparable to that observed for the strain 1-4a in this study (Wei et al., 2013; Xu et al., 2016a). The significant differences in adhesion and invasion efficiencies found between the two *S. zooepidemicus* strains in our study correspond to levels of strain variable invasion efficiencies previously observed for *S. pyogenes* and *S. suis* (Molinari and Chhatwal, 1998; Benga et al., 2004).

Both *S. zooepidemicus* strains investigated showed intracellular survival. In a previous *in vitro* study we have demonstrated that the same strains, when incubated in broth, can form persister cells that survive transient exposure to penicillin (Petersen et al., 2015). Persister cells consist of a small subpopulation of the normal growing population which have switched into a dormant phenotype (Kim and Wood, 2016). Dormancy makes persister cells not only multidrug tolerant, but also able to survive different stress factors like low pH, which they will often encounter in the intracellular environment, and nutrient starvation, as found in biofilms (Maisonneuve and Gerdes, 2014). In the previous study the persister levels varied according to the growth phase, with recovery of 4% of strain 1-4a and 23% of strain S31A1 when the strains were in late exponential phase after having been exposed to penicillin for 24 h (Petersen et al., 2015). We have performed time-kill curves for the strains showing no significant reduction in the populations within the first 6 h of penicillin exposure and recovery of on average 0.01% of strain 1-4a and 0.1% of strain S31A1 after 48 h of penicillin exposure when challenged during stationary phase of growth (data not shown). These results indicated that strain S31A1 forms persister cells to a higher extend than 1-4a *in vitro*. We speculate, based on the present study, that strain 1-4a survival was primarily due to intracellular survival, which might also include formation of persister cells, while strain S31A1 demonstrated low invasiveness, but have a higher persister cell level, which would result in both intra- and extracellular survival, possibly linked to higher expression of pili promoting co-aggregation and biofilm formation. Recurrent infections and carrier stages are recognized in other streptococcal species, e.g., *S. pyogenes* has shown intracellular survival in epithelial cells with reduced killing by penicillin (Kaplan et al., 2006) and lack of clearance *in vivo* despite penicillin treatment (Kaplan et al., 1981; Rohde and Cleary, 2016). For the strict equine pathogen *S. equi* subsp. *equi* persistent carrier state is recognized (Harris et al., 2015), but whether this is due to intracellular persistence is not clear. Intracellular pathogens are notoriously hard to treat and often give rise to chronic infections (Monack et al., 2004), which might be due to a combination of the pathogen having evolved strategies to avoid intracellular degradation, as e.g., *Salmonella, Legionella*, and *Chlamydia* by affecting the endosome or phagosome, hampering the maturation and avoiding fusion with lysosomes (Cossart and Helenius, 2014), and the indications that the intracellular state in itself promotes persister cell formation, as seen for experimental infections of mice with *Salmonella* Typhimurium (Helaine et al., 2014). Demonstrating infections by persister cells *in vivo* is a complicated task due to the very small amount of bacterial cell and their innate nature. Also, additional aspects other than intracellular invasion likely play an important role, such as, the host immune status, level of bacterial biofilm formation, and antimicrobial resistance.

In conclusion we have demonstrated that *S. zooepidemicus* was able to invade human epithelial cells through different uptake mechanisms. The two strains investigated adhered and invaded at significantly different magnitudes. Both strains were found intracellularly after 23 h in compartments rarely associated with lysosomes. The results indicate that survival in an intracellular milieu can be part of *S. zooepidemicus* pathogenesis, possibly together with streptococcal persister cell formation, and that these aspects should be considered when faced with recurrent *S. zooepidemicus* infections *in vivo*. Future studies should focus on identifying the molecular mechanisms governing internalization and intracellular trafficking. Also, further studies using equine endometrial cells isolated from the endometrium of mares would contribute to confirm the biological relevance of our findings. *In vivo* studies should clarify if an intracellular state can be demonstrated in the mare.

## Author contributions

BS: Substantial contributions to the conception and design of all the work, and the acquisition, analysis, and interpretation of data for the work; and drafting, final approval and agreement to be accountable for all aspects of the work. MR: Substantial contributions to the design and acquisition of the work and interpretation of data; revising it critically, and final approval and agreement to be accountable for all aspects of the work. GM: Substantial contributions to the design and interpretation of data; revising it critically, and final approval and agreement to be accountable for all aspects of the work. TB: Substantial contributions to the acquisition and analysis and interpretation of data; revising it critically, and final approval and agreement to be accountable for all aspects of the work. AB: Substantial contributions to the conception and interpretation of data for the work; and drafting, final approval and agreement to be accountable for all aspects of the work.

### Conflict of interest statement

The authors declare that the research was conducted in the absence of any commercial or financial relationships that could be construed as a potential conflict of interest.
